# Management of Diffuse Large B-Cell Lymphoma as Post-Transplant Lymphoproliferative Disorder in a Kidney Transplant Recipient: A Case Report

**DOI:** 10.3390/hematolrep17030022

**Published:** 2025-04-23

**Authors:** Salem Alshemmari, Abdulaziz Hamadah, Samar Ousia, Rasha Abdel Tawab Hamed, Hany Zaky

**Affiliations:** Oncology/Hematology Department, Kuwait Cancer Control Center, Kuwait City 70653, Kuwait; ahamadah@moh.gov.kw (A.H.); sousia@moh.gov.kw (S.O.); rabdeltawab@moh.gov.kw (R.A.T.H.); hkhedr@moh.gov.kw (H.Z.)

**Keywords:** loncastuximab tesirine, post-transplant lymphoproliferative disorder, diffuse large B-cell lymphoma

## Abstract

**Background and Clinical Significance:** Post-transplant lymphoproliferative disorder (PTLD) is a severe complication of solid organ transplantation, often associated with prolonged immunosuppression. Diffuse large B-cell lymphoma (DLBCL) is the most common subtype. Managing PTLD requires a balance between reducing immunosuppression and preventing graft rejection. **Case Presentation**: A 41-year-old female kidney transplant recipient developed PTLD eight years post-transplant, presenting with a right submandibular mass. Biopsy confirmed CD20-positive DLBCL. Initial treatment involved reducing immunosuppression and rituximab monotherapy, which failed to prevent disease progression. The patient underwent six cycles of R-CHOP chemotherapy, achieving complete metabolic remission. Relapse occurred twice, with disease progression in the cervical nodes and tonsils. Salvage therapies, including polatuzumab vedotin and rituximab, achieved remission. During a subsequent relapse, loncastuximab tesirine induced metabolic resolution. Compromised renal function limited treatment options and a second renal transplant was delayed, reducing the risk of PTLD recurrence. **Conclusions**: This case underscores the challenges of managing PTLD in transplant recipients, especially in relapsed/refractory cases. Single-agent rituximab was insufficient, but combination chemotherapy and novel agents like loncastuximab tesirine were effective. Balancing oncologic control and graft preservation remains critical. This case highlights the need for individualized approaches and novel therapies in managing PTLD while addressing the complexities of immunosuppression and organ preservation.

## 1. Introduction

Post-transplant lymphoproliferative disorder (PTLD) is a significant and potentially life-threatening complication following solid organ transplantation, arising due to prolonged immunosuppression that impairs normal immune surveillance mechanisms [1]. PTLD includes a spectrum of lymphoid proliferations ranging from benign hyperplasias to aggressive lymphomas, with diffuse large B-cell lymphoma (DLBCL) being the most common subtype [2]. The incidence of PTLD varies among transplant recipients but is notably higher in those who have received kidney transplants, with an estimated risk increase of up to 20-fold compared to the general population [3].

The management of PTLD poses a clinical challenge, as it requires a delicate balance between reducing immunosuppression to control the lymphoproliferative disorder and maintaining sufficient immunosuppression to prevent graft rejection [4]. Initial therapeutic strategies often involve the reduction or modification of immunosuppressive therapy, but this approach alone may be insufficient, especially in aggressive cases [5]. Rituximab, a monoclonal anti-CD20 antibody, has been employed as monotherapy or in combination with chemotherapy regimens such as CHOP (cyclophosphamide, doxorubicin, vincristine, prednisone) with varying degrees of success [6].

Recent advancements in targeted therapies have introduced novel agents like polatuzumab vedotin and loncastuximab tesirine, which have shown efficacy in relapsed or refractory DLBCL [7,8]. These agents offer alternative mechanisms of action by delivering cytotoxic agents directly to malignant B cells, thereby potentially reducing systemic toxicity. This case report examines a 41-year-old female kidney transplant recipient who developed DLBCL as a form of PTLD. It aims to highlight the complexities of managing PTLD in transplant patients, particularly in adjusting immunosuppressive treatments and preserving renal function. The report discusses various therapeutic interventions, including traditional chemotherapy, monoclonal antibodies, and newer antibody–drug conjugates.

## 2. Case Presentation

A 41-year-old Kuwaiti woman presented with a complex medical history, including hypertension and systemic lupus erythematosus (SLE), diagnosed in 2006. Her SLE was complicated by lupus nephritis, leading to end-stage renal disease and requiring regular haemodialysis since 2011. She underwent a live renal transplant in 2012, with her father as the donor. Post-transplant, she was maintained on prednisolone 10 mg daily along with tacrolimus and mycophenolate mofetil as part of her immunosuppression regimen.

**Figure 1:**
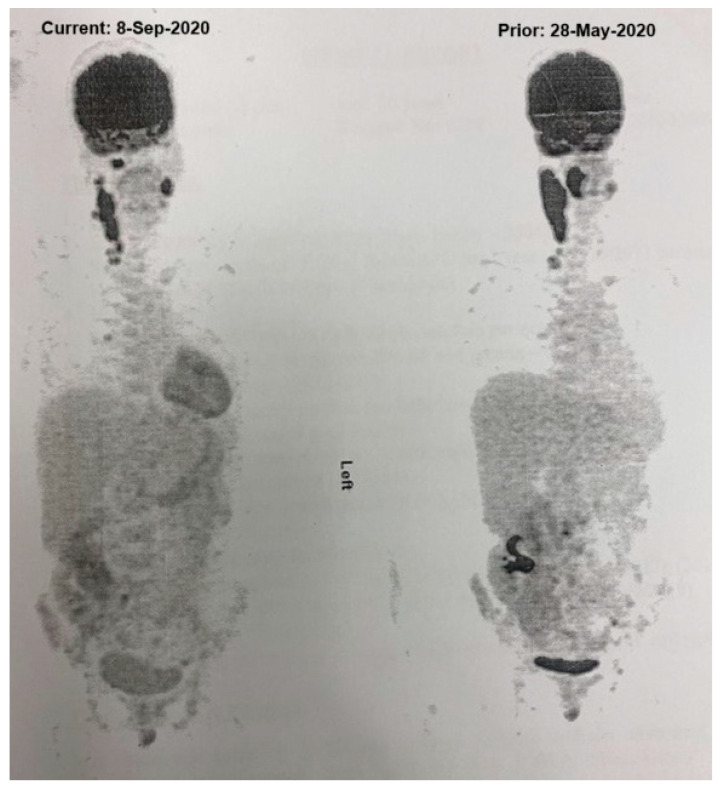
PET Scan Findings Indicating Disease Progression in PTLD; PET scan performed on 13 September 2020, showing evidence of disease progression with the appearance of a new hypermetabolic right retropharyngeal lymph node (SUV max 13.5) and an interval increase in metabolic activity in cervical and pelvic lymph nodes, consistent with progressive PTLD. This image reflects the initial presentation and the first relapse.

In April 2020, she developed a swelling in the right submandibular region that progressed to a 5 × 5 cm mass over several weeks. Imaging studies, including a computed tomography (CT) scan performed in June 2020, revealed a hypopharyngeal mass with bilateral lymphadenopathy, confirmed by a magnetic resonance imaging (MRI) study later that month. A positron emission tomography (PET) scan conducted in May 2020 showed a hypermetabolic conglomerate mass in the right neck and oropharynx with a maximum SUV of 23.1, as well as suspicious para-aortic and obturator lymph nodes Figure 1). An initial fine needle aspiration cytology (FNAC) suggested granulomatous lymphadenitis. Due to persistent suspicion, a repeat FNAC was performed, revealing suspicious cytology. A tonsillar excision biopsy in July 2020 confirmed DLBCL, with tumour cells positive for CD20 and a Ki-67 index of 80%. Fluorescence In Situ Hybridization (FISH) analysis performed on the initial biopsy specimen revealed the absence of Myc, BCL-2, and BCL-6 rearrangements.

Laboratory investigations revealed significant abnormalities, including leukocytosis with a white blood cell (WBC) count of 40.0 × 10^9^/L, anaemia with haemoglobin (Hb) at 92 g/L, and thrombocytosis with a platelet (PLT) count of 174 × 10^9^/L. Renal function was impaired, as indicated by elevated creatinine levels at 302 µmol/L and an estimated glomerular filtration rate (eGFR) of 15 mL/min/1.73 m^2^. Cytomegalovirus (CMV) polymerase chain reaction (PCR) titre was elevated at 895 IU/mL, while testing for Epstein–Barr virus (EBV) was unavailable. Screening for hepatitis B and C viruses, as well as human immunodeficiency virus (HIV), returned negative results. These findings, along with clinical and imaging data, confirmed the diagnosis of PTLD.

Initial management involved reducing immunosuppression, which included discontinuing mycophenolate mofetil while continuing tacrolimus at a reduced dose. The patient received four doses of rituximab during August 2020, starting with 600 mg intravenously, followed by three weekly subcutaneous doses of 1400 mg. Concurrently, she was treated with valganciclovir 450 mg every other day for CMV reactivation, guided by her renal function. CMV antigenemia resolved by mid-August 2020, and valganciclovir was discontinued. Despite these measures, a follow-up PET scan in September 2020 showed disease progression with new hypermetabolic lesions in the right retropharyngeal lymph node and increased metabolic activity in existing cervical and pelvic nodes (Figure 1). Consequently, she was started on R-CHOP chemotherapy on 1October 2020. An interim PET scan after four cycles in November 2020 revealed a complete metabolic response, and she completed six cycles by January 2021. A PET scan in February 2021 confirmed sustained remission.

In October 2021, the patient developed a hard palate mass that progressively enlarged and infiltrated the mucosa. A biopsy revealed recurrent DLBCL, with tumour cells positive for CD20, CD79a, and Bcl-6, and a Ki-67 index of approximately 80%. The case was discussed in a multidisciplinary team (MDT) meeting, and salvage chemotherapy with rituximab (375 mg/m^2^), polatuzumab vedotin (1.8 mg/kg), and bendamustine (90 mg/m^2^) was initiated in November 2021. The patient completed six cycles by May 2022, with a PET scan in July 2022 confirming complete metabolic response (Table 1). She remained in remission under close follow-up.

**Figure 2:**
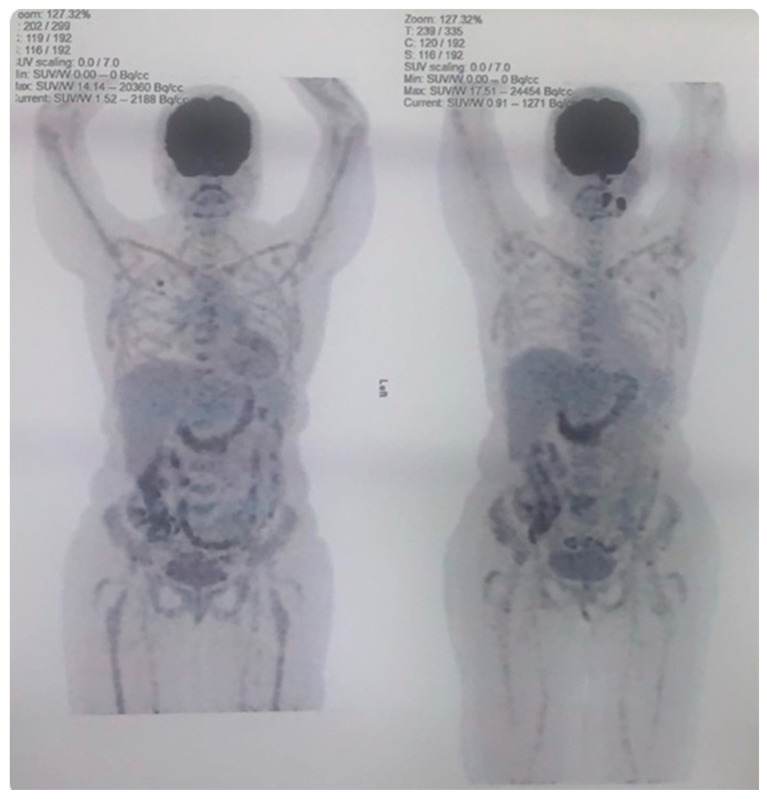
Interim PET Scan Demonstrating Metabolic Resolution in PTLD; PET scan conducted on 20 October 2024, showing interval metabolic resolution of previously hypermetabolic lesions in the left cervical lymph node, tonsillar region, left scapula, and right iliac bone, indicating treatment response in PTLD. The patient subsequently received her fifth cycle of therapy on 24 October 2024. This image reflects the response to loncastuximab tesirine.

In March 2024, the patient experienced another relapse, presenting with left cervical lymphadenopathy and tonsillar involvement. Epstein–Barr virus (EBV) testing was not performed at the time of relapse as the test was unavailable. A PET scan showed hypermetabolic lesions with Focal 18 F-fluorodeoxyglucose (FDG) uptake in the scapula and iliac bone. A biopsy confirmed high-grade non-Hodgkin lymphoma. The MDT recommended initiating loncastuximab tesirine due to poor renal function (serum creatinine 633 µmol/L, eGFR < 10 mL/min/1.73 m^2^). The patient received four cycles of loncastuximab tesirine, with a dosing regimen consisted of 0.15 mg/kg for the first two cycles, followed by 0.075 mg/kg for subsequent cycles. In October 2024, the patient achieved a metabolic response as evidenced by an interim PET scan. She has since received a fifth cycle and remains under close observation (Figure 2).

## 3. Discussion

The management of PTLD in solid organ transplant recipients presents significant clinical challenges, particularly in balancing effective oncologic treatment with the preservation of graft function [9]. This case of a 41-year-old female kidney transplant recipient who developed DLBCL as a manifestation of PTLD highlights these complexities and offers insights into therapeutic strategies and outcomes. Upon diagnosis of DLBCL, the patient’s immunosuppressive regimen was modified by discontinuing mycophenolate mofetil and reducing the dose of tacrolimus. This approach aligns with standard practices aimed at enhancing immune surveillance to control lymphoproliferation while minimizing the risk of graft rejection [10,11]. However, the efficacy of immunosuppression reduction alone is often limited, especially in aggressive PTLD cases, necessitating additional therapeutic interventions [12,13]. It was reported that only 10% of PTLD patients significantly respond to immunosuppressant reduction [14,15].

The administration of rituximab, an anti-CD20 monoclonal antibody, as initial therapy reflects its established role in treating CD20-positive PTLD. In two large, multicentre phase II studies, weekly doses of rituximab (375 mg/m² for four weeks) achieved complete remission rates of 52% and 28%, with overall response rates of 44% to 50% [14,15]. However, combined analysis showed a high progression rate, with 50% of patients experiencing disease progression within six months and 57% within a year [16]. Although initial responders (57%) showed some durable benefit, many required additional therapies, including rituximab retreatment, chemotherapy, or irradiation, leading to overall survival rates of 72.5% and 51.8% at one and two years, respectively [16]. Extended rituximab regimens, as explored in a Spanish trial, improved complete remission rates to 61%, though the impact on overall survival remains uncertain [17]. Furthermore, a retrospective analysis demonstrated that selecting low-risk patients for rituximab monotherapy yielded promising results, with 76% achieving long-term remission, emphasizing its efficacy in appropriately chosen patient populations [18]. Similarly, in this case, disease progression observed on follow-up imaging indicated that rituximab alone was insufficient, prompting the initiation of combination chemotherapy.

The patient received six cycles of R-CHOP, resulting in a complete metabolic response confirmed by PET scans. This outcome is consistent with the existing literature where R-CHOP has been effective in achieving remission in PTLD patients [19]. However, the regimen’s associated toxicities and immunosuppressive effects necessitate careful patient monitoring, especially in transplant recipients. The subsequent relapse with a hard palate mass highlights the aggressive nature of PTLD and the challenges in achieving sustained remission. Salvage therapy with polatuzumab vedotin, bendamustine, and rituximab was employed, leading to another complete metabolic response. Polatuzumab vedotin, an antibody–drug conjugate targeting CD79b, has shown efficacy in relapsed/refractory DLBCL, offering a therapeutic option for patients who have failed prior treatments [20,21].

The patient’s second relapse, characterized by cervical lymphadenopathy and tonsillar involvement, was managed with loncastuximab tesirine, a CD19-directed antibody–drug conjugate. This agent has demonstrated substantial single-agent efficacy in heavily pretreated patients with relapsed or refractory DLBCL, including those with high-risk features [7,22,23]. In this case, administration of loncastuximab tesirine resulted in a metabolic response, as evidenced by interim PET scans.

In the context of relapsed or refractory DLBCL, high-dose chemotherapy followed by autologous stem cell transplantation (ASCT) is a well-established therapeutic strategy [24]. However, the eligibility for such intensive treatment is contingent upon the patient’s overall health status and comorbid conditions [25]. Our patient, with a history of systemic lupus erythematosus complicated by end-stage renal disease and ongoing hypertension, presented a high-risk profile for treatment-related complications. The choice of treatment following each relapse was guided by the patient’s clinical condition and available therapeutic options. After the first relapse, high-dose chemotherapy and ASCT were not considered due to her compromised renal function and overall treatment-related risk profile. Instead, we opted for the polatuzumab vedotin, rituximab, and bendamustine (Pola-RB) protocol, as it is a less intensive regimen with demonstrated efficacy in relapsed/refractory DLBCL. At the time of the second relapse, the patient’s renal impairment had further deteriorated, restricting the use of certain therapies. Chimeric antigen receptors T-cell (CAR-T) therapy was considered; however, it was not pursued due to the unavailability of the treatment locally. Given these constraints, treatment decisions were made to balance efficacy and tolerability while preserving renal function. In the event of a further relapse, alternative treatment options remain limited due to the patient’s comorbidities and renal impairment. However, we are currently exploring the possibility of arranging CAR-T cell therapy in a nearby country to expand the landscape of therapeutic options should the disease progress again.

Throughout the treatment course, the patient’s renal function remained a critical concern, particularly given her history of lupus nephritis and end-stage renal disease. The decision to delay a second renal transplant underscores the complexity of managing PTLD in patients with compromised renal function, where aggressive lymphoma treatment must be balanced against the potential for nephrotoxicity and the need for ongoing immunosuppression.

This case is unique due to the use of loncastuximab tesirine in the treatment of relapsed/refractory PTLD. Given the patient’s severe renal impairment and ineligibility for high-dose chemotherapy or stem cell transplantation, loncastuximab tesirine was selected for its targeted mechanism and tolerable toxicity profile, leading to a metabolic response. While its use in PTLD is not well-documented, this case highlights its potential as an alternative therapy for high-risk patients. Future research should explore its safety and long-term efficacy in transplant recipients with refractory disease. On the other hand, this case is limited by the lack of comprehensive EBV investigations. Future studies should consider performing an EBV assay or a tonsil excisional biopsy to confirm the presence or absence of EBV.

Additionally, this case highlights the complexities of managing PTLD in kidney transplant recipients, particularly in the setting of multiple relapses and significant comorbidities. The patient’s treatment course highlights the importance of an individualized, multidisciplinary approach that balances oncologic control with immunosuppressive management and organ preservation. The use of novel therapies, such as polatuzumab vedotin and loncastuximab tesirine, demonstrates that targeted treatments can provide effective disease control in patients who are ineligible for high-dose chemotherapy or ASCT. Future management strategies for similar cases should incorporate early molecular profiling, including cell-of-origin classification and EBV status, to guide personalized treatment decisions.

Further research is needed to optimize treatment algorithms for relapsed/refractory PTLD, particularly in transplant recipients with impaired renal function. Prospective studies should evaluate the long-term safety and efficacy of novel immunotherapies, including bispecific T-cell engagers (BiTEs) and CAR-T cell therapy, in this high-risk patient population. Additionally, real-world data on treatment sequencing, renal toxicity profiles, and graft survival outcomes in patients receiving novel lymphoma therapies would be valuable for refining clinical guidelines. Expanding access to advanced therapeutics and establishing standardized approaches to integrating targeted therapies into PTLD management should be key priorities for future research.

In conclusion, this case highlights the multifaceted challenges in managing PTLD in kidney transplant recipients, including the need for individualized treatment strategies that consider both oncologic control and graft preservation. The successful use of novel therapies such as loncastuximab tesirine offers promising avenues for patients with relapsed or refractory disease, though further studies are warranted to establish their efficacy and safety in this unique patient population.

## Figures and Tables

**Table 1:** Summary of treatment and outcomes.

Treatment Regimen	Timeframe	Outcome
Immunosuppression Reduction	April 2020	Discontinued mycophenolate mofetil, reduced tacrolimus
Rituximab Monotherapy (4 doses)	August 2020	Disease progression (PET scan: new hypermetabolic lesions)
R-CHOP Chemotherapy (6 cycles)	October 2020–January 2021	Complete metabolic remission (February 2021 PET scan)
Salvage Therapy (Polatuzumab vedotin + Rituximab + Bendamustine, 6 cycles)	November 2021–May 2022	Complete metabolic remission (July 2022 PET scan)
Loncastuximab Tesirine (4 cycles)	March–October 2024	Metabolic resolution (October 2024 PET scan)

## Data Availability

The raw data supporting the conclusions of this article will be made available by the authors on reasonable request.
